# The interplay between stroke and hip fractures: a narrative review of mechanisms, risk assessment, and prevention

**DOI:** 10.3389/fragi.2026.1780462

**Published:** 2026-04-09

**Authors:** Zuzhou Wen, Yun Ye, Junzhao Qiao, Hongqiang Chen

**Affiliations:** 1 Department of Joint Orthopedics, The Fourth People’s Hospital of Guiyang, Guiyang, Guizhou, China; 2 Bone Diseases & Center for Healthy Aging,University Hospital Carl Gustav Carus of the Technische Universität Dresden, Dresden, Germany

**Keywords:** fracture risk, functional impairment, hip fracture, mortality, rehabilitation management, stroke

## Abstract

Stroke survivors face a substantially increased risk of hip fractures due to a combination of motor impairments, falls, and stroke-induced bone density loss. Conversely, hip fractures in this population are associated with elevated mortality, reduced functional recovery, and greater healthcare burden. This narrative review synthesizes current evidence on the epidemiological, pathophysiological, and clinical interrelationships between stroke and hip fractures. Key quantitative findings include a 7.6% incidence of hip fractures within 5 years post-stroke—significantly higher than the 2.8% observed in non-stroke populations—and a 30-day postoperative mortality rate of up to 14.8% in stroke patients with hip fractures. Severe post-stroke disability is associated with a 2.1- to 4.8-fold increased risk of hip fracture compared to those with good recovery. The review also highlights the utility of risk assessment tools such as FRAX and emerging prediction models, and evaluates prevention strategies including osteoporosis screening, exercise-based rehabilitation, and lifestyle modifications.

## 1 Introduction

Stroke is a leading cause of disability and mortality worldwide, and its impact is particularly pronounced among older adults. The consequences of stroke extend beyond immediate health concerns, significantly affecting patients’ quality of life and increasing the risk of subsequent health complications. The risk of hip fractures increases after a stroke, because stroke survivors may experience mobility impairments and osteoporosis, both of which can increase their vulnerability to fractures.

One of the most alarming aspects of stroke is its association with long-term functional impairments. Stroke survivors frequently face challenges in mobility and balance, leading to an increased risk of falls and subsequent hip fractures. For instance, studies have shown that individuals with severe post-stroke disabilities are at a significantly higher risk of experiencing hip fractures compared to those with mild disabilities or good recovery status (Northuis et al., 2020). Furthermore, the presence of osteoporosis in stroke survivors exacerbates the risk of fracture, as the loss of bone density is a common consequence of immobility and the physiological changes following a stroke (Hsieh et al., 2020). The combined effect of these factors contributes to a detrimental cycle in which hip fractures lead to further health deterioration, increased mortality, and a decline in the overall quality of life for stroke survivors.

Hip fractures not only represent a physical challenge for stroke patients but also carry significant implications for healthcare systems. The management of hip fractures in this population is complex, often requiring surgical intervention and extensive rehabilitation. Studies have shown that mortality after hip fractures is alarmingly high, especially in older adults with comorbidities (Hjelholt et al., 2022). The economic burden associated with hip fractures is substantial, with estimates suggesting that the costs related to hospitalization and rehabilitation can reach billions annually (Yang et al., 2021). This economic impact underscores the need for effective prevention strategies to mitigate the risk of hip fractures in stroke patients.

Recent research has begun to shed light on the specific risk factors that contribute to the heightened incidence of hip fractures among stroke survivors. Factors such as age, gender, and pre-existing health conditions play a pivotal role in determining fracture risk (Zhang et al., 2023; Lee J. H. et al., 2024). Although there is a growing body of literature on the relationship between stroke and hip fractures, a notable gap remains in narrative reviews that synthesize findings and provide actionable insights for clinical practice. This narrative review aims to fill that gap by analyzing the latest evidence on the epidemiology of hip fractures in stroke patients, identifying key risk factors, and evaluating current prevention strategies. By leveraging recent studies and data, we hope to offer a framework for clinicians to enhance patient outcomes and reduce the incidence of hip fractures in this vulnerable population.

## 2 Methods

A comprehensive literature search was conducted to identify studies examining the relationship between stroke and hip fractures, with a focus on mechanisms, risk assessment, prevention, and clinical outcomes. The search was performed in accordance with the PRISMA (Preferred Reporting Items for Systematic Reviews and Meta-Analyses) guidelines to ensure transparency and reproducibility. We systematically searched the following electronic databases: PubMed/Medline and Web of Science. The search was limited to studies published in English. No restriction on publication date was applied, but priority was given to articles published within the last 10 years to capture the most recent evidence. Animal studies, case reports, editorials, and commentaries were excluded. In addition, we manually screened the reference lists of included articles and relevant review papers to identify additional eligible studies. Studies were included if they met the following criteria: (1) original research (cohort, case–control, cross-sectional, or randomized controlled trials) or systematic reviews/meta-analyses; (2) involved adult patients (≥18 years) with a diagnosis of stroke; (3) reported data on hip fracture incidence, risk factors, prediction models, prevention strategies, or outcomes following hip fracture in stroke populations; (4) provided sufficient quantitative or qualitative data for synthesis. Studies that did not specifically address the link between stroke and hip fractures, or that focused exclusively on other types of fractures, were excluded. Eventually, after reading the full texts 89 articles were included based on the quality of the studies.

## 3 Epidemiological characteristics of hip fractures after stroke

### 3.1 Incidence and temporal distribution of hip fractures in stroke patients

Recent studies have highlighted a concerning trend regarding the incidence of hip fractures in patients who have experienced a stroke. Specifically, the occurrence of hip fractures in stroke patients within 5 years post-stroke is significantly elevated compared with non-stroke populations (7.6% versus 2.8%) (Salehi Omran et al., 2020). This stark difference highlights the urgent need for targeted preventive strategies in this vulnerable group. Furthermore, the heightened risk of hip fractures in the early years following a stroke indicates that the first few months after stroke are critical for intervention. Research shows that this increased risk is likely due to a combination of factors. These include impaired mobility, balance issues, and potential cognitive deficits that affect the patient’s ability to engage in safe physical activities (Chen et al., 2024). Moreover, the relationship between stroke severity, as measured by the NIHSS score, and the likelihood of sustaining a hip fracture further complicates the clinical picture. Patients with more severe strokes tend to experience greater muscle weakness and sensory impairments, which can exacerbate their risk of falls and subsequent hip fractures (Wang et al., 2021).

In light of these findings, it is imperative that healthcare providers implement comprehensive risk assessment protocols for stroke patients, particularly focusing on fall risk and bone health. Early interventions, such as physical therapy aimed at improving balance and strength, should be prioritized during the rehabilitation process to mitigate the risk of hip fractures. Additionally, regular screenings for osteoporosis and nutritional assessments to ensure adequate calcium and vitamin D intake could play a crucial role in fracture prevention among this demographic. The integration of these strategies into post-stroke care plans could significantly enhance patient outcomes and reduce the burden of hip fractures in this high-risk population.

### 3.2 Differences in incidence among different populations and regions

Stroke incidence and its associated complications, such as hip fractures, exhibit significant variability across different populations and geographical regions. This variability is influenced by factors including gender, age, race, and socioeconomic status. For instance, studies indicate that men generally have a higher incidence of stroke than women. However, post-stroke complications such as hip fractures have a higher incidence and more severe consequences in women, likely due to differences in bone density and osteoporosis prevalence (Talbot et al., 2022). Age is another critical factor, with older adults, particularly those over 65, experiencing a disproportionate burden of both stroke and subsequent hip fractures (Austin et al., 2024). Ethnic disparities are also evident; for example, white populations in certain countries have been reported to have a higher risk of stroke compared to other racial groups, which may be influenced by genetic predispositions and lifestyle factors (Marquez-Romero et al., 2024). Geographic differences add complexity to this picture, as studies show that stroke incidence is markedly higher in regions such as North America compared to Asia and Europe (Lee et al., 2022; Youens et al., 2023).

Moreover, specific risk factors such as diabetes, hypertension, and obesity have been shown to have a more pronounced effect on increasing stroke incidence in certain populations. For example, the association between diabetes and stroke risk is particularly strong in low- and middle-income countries, where the prevalence of diabetes is rising rapidly (Yao et al., 2023). In contrast, in high-income countries, the focus has shifted towards managing hypertension and lifestyle factors as primary preventive measures against stroke.

Meanwhile, socioeconomic factors play a critical role in stroke incidence and outcomes. Populations living in areas with limited access to healthcare services or lower socioeconomic status have been found to have higher rates of stroke and associated complications, including hip fractures (Tan et al., 2020). This disparity highlights the need for targeted public health interventions that consider both individual and community-level factors to effectively reduce the incidence of stroke and its complications across diverse populations.

In summary, understanding the differences in stroke incidence among various populations and regions is crucial for developing effective prevention and management strategies. Factors such as gender, age, race, and socioeconomic status must be considered to tailor interventions that address the unique needs of different communities, ultimately aiming to reduce the burden of stroke and its complications, including hip fractures.

## 4 Pathophysiological foundations of hip fracture risk after stroke

### 4.1 Motor dysfunction and increased risk of falls

Stroke is a significant contributor to motor dysfunctions—such as balance deficits, muscle weakness, and cognitive decline—that profoundly impact an individual’s functional independence and quality of life. These motor impairments significantly increase the risk of falls among stroke survivors. Research indicates that post-stroke patients often experience specific motor dysfunctions, which can be quantitatively assessed using tools such as the Berg Balance Scale (BBS). This scale has been shown to correlate strongly with the risk of falls, particularly in individuals with severe disabilities, suggesting that those with lower BBS scores are at a heightened risk for both falls and subsequent fractures (Zhu et al., 2025; Wang et al., 2023). The underlying mechanisms contributing to this increased fall risk are multifaceted. For instance, stroke can lead to impaired proprioception and vestibular function, both crucial for maintaining balance during ambulation. Additionally, muscle weakness—particularly in the lower extremities—further exacerbates instability and increases susceptibility to falls (Shi et al., 2023; Oquita et al., 2024).

The relationship between motor dysfunction and falls is further complicated by cognitive impairments that frequently accompany stroke. Cognitive deficits can impair an individual’s ability to process environmental information, make quick decisions, and execute timely motor responses, all essential for maintaining balance and preventing falls. Studies have shown that cognitive dysfunction is associated with increased intraindividual variability in motor performance, which can serve as an indicator of fall risk (Delmas et al., 2025). Furthermore, the fear of falling, prevalent among stroke survivors, can lead to reduced physical activity and deconditioning, creating a vicious cycle that further increases fall risk (Zhang et al., 2025; Ozkan et al., 2024).

The evidence suggests that interventions targeting motor and cognitive functions may effectively reduce fall risk in stroke patients. Rehabilitation strategies incorporating balance training, strength exercises, and cognitive rehabilitation have shown promise in enhancing functional outcomes and reducing fall incidence (Cai et al., 2024; Fu et al., 2024). For instance, programs utilizing task-oriented training and dual-tasking exercises can improve both motor performance and cognitive function, thereby mitigating fall risk (Shen et al., 2025). Additionally, implementing comprehensive fall risk assessments in clinical settings can aid in identifying individuals at high risk and tailoring interventions accordingly (Wan et al., 2025).

In summary, the interplay between motor dysfunction and increased fall risk in stroke survivors necessitates a multifaceted approach to rehabilitation. By addressing both the physical and cognitive dimensions of recovery, healthcare providers can better support stroke patients in regaining functional independence and reducing the likelihood of falls and associated injuries.

### 4.2 Osteoporosis and abnormal bone metabolism

Stroke survivors often experience a marked reduction in bone mineral density (BMD), significantly increasing their risk of osteoporosis and subsequent fractures. This decline is driven by a complex interplay of physiological and biochemical mechanisms triggered by the cerebrovascular event. A primary factor is the disruption of neural pathways that regulate bone metabolism, leading to reduced mobility and mechanical unloading. According to the mechanostat theory, this lack of weight-bearing activity diminishes osteoblast-mediated bone formation while allowing osteoclastic resorption to proceed unopposed, resulting in a net loss of bone mass and structural integrity (Gao et al., 2022). This process is further compounded by a neuroinflammatory response, characterized by the release of pro-inflammatory cytokines such as interleukin-6 (IL-6) and tumor necrosis factor-alpha (TNF-α). These molecules not only stimulate osteoclastogenesis but also inhibit osteoblast differentiation and activity, thereby accelerating bone loss (Shi, 2021; Orassi et al., 2021).

In addition to these mechanical and inflammatory pathways, significant hormonal changes play a critical role. The acute stress of a stroke can dysregulate the hypothalamic-pituitary-adrenal (HPA) axis, leading to elevated cortisol levels. Cortisol is known to suppress osteoblast function and promote osteoclast activity, exacerbating the imbalance in bone turnover (Sun et al., 2023; Werner et al., 2022). Furthermore, the use of certain post-stroke medications, such as glucocorticoids, can independently contribute to skeletal deterioration by inhibiting bone formation and promoting resorption (Zou et al., 2025). This already compromised state is often worsened by the presence of common comorbidities like pre-existing osteoporosis, diabetes, or chronic kidney disease, which can independently alter bone metabolism and increase fragility (Trojniak et al., 2024; Liu et al., 2021). Given these multifactorial contributions to bone loss, effective clinical management for stroke survivors necessitates a comprehensive approach. This should include regular assessments of bone health, targeted physical rehabilitation to encourage weight-bearing activity, nutritional optimization with calcium and vitamin D, and the consideration of pharmacological interventions like bisphosphonates for high-risk patients to mitigate fracture risk (Singer, 2021; Fereidoonnezhad et al., 2021; Borges et al., 2024).

### 4.3 Other influencing factors

The risk of hip fractures in patients who have suffered a stroke is significantly influenced by various factors, including smoking habits and comorbid conditions such as hypertension and diabetes. Both ongoing and newly initiated smoking have been identified as critical risk factors that further increase the likelihood of fractures. Studies indicate that smokers are at a higher risk for osteoporosis and subsequent fractures due to the detrimental effects of nicotine on bone density and overall skeletal health. For instance, nicotine has been shown to impair osteoblast function, which is essential for bone formation, thereby increasing the risk of fractures in individuals with a history of stroke (Lee J. H. et al., 2024; Xie et al., 2024). Furthermore, the interplay between smoking and other lifestyle factors, such as physical inactivity, further elevates the risk, particularly in the elderly population who may already be vulnerable due to age-related bone density loss.

In addition to smoking, the presence of comorbidities such as hypertension and diabetes significantly heightens the risk of hip fractures post-stroke. Hypertension, a prevalent condition among stroke survivors, is associated with vascular changes—such as arterial stiffness—that can lead to compromised blood flow to bones, thereby increasing fragility and fracture risk. Elevated blood pressure has been linked to increased bone resorption and decreased bone formation, both of which are critical processes in maintaining bone health (Xie et al., 2024). In a similar vein, diabetes, particularly type 2 diabetes, has been associated with an increased risk of fractures due to factors such as neuropathy, which can lead to falls, and the adverse effects of hyperglycemia on bone quality.

The cumulative effect of these risk factors underscores the importance of comprehensive risk assessment and management strategies in stroke survivors. Effective management of hypertension and diabetes, combined with smoking cessation programs, may significantly reduce the risk of hip fractures in this vulnerable population. Moreover, healthcare providers should be vigilant in monitoring these patients for signs of osteoporosis and consider implementing preventive measures, such as bone density screenings and lifestyle modifications, to enhance bone health. By addressing these modifiable risk factors, the burden of hip fractures in stroke survivors can be reduced, ultimately improving their quality of life and decreasing healthcare costs associated with fracture management.

## 5 The association between stroke disability severity and hip fracture risk

### 5.1 Significantly increased fracture risk in severely disabled patients

Severe disability following a stroke is a critical factor influencing the risk of subsequent hip fractures, with studies indicating that the fracture risk in these patients is more than double that of individuals who achieve good recovery. For instance, a study found that patients with severe disability had a hazard ratio (HR) of 2.1 for hip fractures compared to those who achieved good recovery, highlighting the stark contrast in fracture risk associated with varying levels of post-stroke disability (Northuis et al., 2020). This increased risk is particularly alarming given that hip fractures are not only prevalent but also associated with significant morbidity and mortality in the elderly population. The underlying mechanisms contributing to this heightened fracture risk include impaired mobility, decreased muscle strength, and an increased risk of osteoporosis, all of which are prevalent in severely disabled stroke survivors. Moreover, the risk of hip fractures is not significantly increased among patients with moderate disability, suggesting that the degree of disability is a crucial predictive factor (Northuis et al., 2020; Liu et al., 2024).

Additionally, a retrospective cohort analysis found that among stroke survivors, those with severe post-stroke disability had a significantly higher incidence of hip fractures, with an adjusted hazard ratio of 4.82 compared to those with mild disability (Lee D. et al., 2024). This finding suggests that interventions aimed at improving mobility and strength in severely disabled patients could be crucial in mitigating the risk of hip fractures. Specifically, tailored rehabilitation programs that focus on enhancing balance, muscle strength, and functional mobility may be particularly effective. The implications of these findings are profound, as they underscore the necessity for targeted fracture prevention strategies in this vulnerable population.

Overall, the evidence clearly indicates that severe disability following a stroke is a significant predictor of increased fracture risk, particularly for hip fractures. Therefore, a multifaceted approach to patient management is necessary. This approach should include regular assessments for osteoporosis, individualized rehabilitation programs designed to enhance mobility and strength, and proactive measures to prevent falls. By addressing these factors, healthcare providers can potentially reduce the incidence of hip fractures among severely disabled stroke survivors, thereby improving their overall quality of life and reducing the burden of disability associated with such injuries.

### 5.2 Clinical application of disability assessment tools

The assessment of disability in patients who have suffered a stroke, particularly in relation to hip fracture risk, is critical for effective clinical management and rehabilitation strategies. Tools such as the Glasgow Outcome Scale (GOS) and the NIH Stroke Scale (NIHSS) are pivotal in evaluating the extent of disability and the subsequent risk of complications, including fractures. The GOS provides a straightforward measure of recovery outcomes in patients with brain injuries, categorizing them into distinct levels of recovery. Meanwhile, the NIHSS quantifies neurological impairment, allowing healthcare providers to gauge the severity of stroke and predict specific recovery patterns, such as motor function improvement and cognitive recovery. Studies have shown that higher NIHSS scores correlate with increased functional impairment and a greater risk of subsequent falls and fractures, particularly hip fractures, which are a significant concern in the aging stroke population (Li et al., 2022; Carcreff et al., 2022).

Furthermore, integrating balance function tests, such as the Berg Balance Scale (BBS), into the assessment framework enhances the predictive capability for fracture risk. The BBS evaluates various aspects of balance and functional mobility, which are crucial in identifying patients at risk of falls. Research indicates that patients with lower BBS scores exhibit a higher incidence of hip fractures post-stroke due to compromised balance and stability (Lu et al., 2024). This multifaceted approach to disability assessment, combining neurological scales with functional tests, allows clinicians to develop tailored rehabilitation plans that address both the immediate and long-term needs of stroke survivors.

In addition to these scales, the incorporation of patient-reported outcome measures (PROMs) into routine assessments can provide valuable insights into the subjective experience of functional impairment, such as perceived difficulties in mobility, self-care, and social participation, further informing clinical decisions. PROMs capture the nuances of how stroke impacts daily living and quality of life, which are often not fully reflected in clinical measures alone (Ibsen et al., 2022). The combination of objective assessments with subjective evaluations creates a comprehensive picture of a patient’s functional status. This enables healthcare providers to implement more effective and personalized interventions aimed at reducing the risk of hip fractures and enhancing overall recovery outcomes.

Furthermore, the ongoing development of digital tools and applications for assessing disability and fracture risk holds promise for improving the efficiency and accuracy of these evaluations. For instance, mobile applications that utilize machine learning algorithms to analyze gait and balance can provide real-time feedback and predictive analytics, thus facilitating timely interventions (Kumar et al., 2025). As the field of rehabilitation continues to evolve, the integration of these advanced assessment tools will be essential in optimizing care for stroke survivors.

In conclusion, the clinical application of disability assessment tools, including the GOS, NIHSS, and balance function tests, is vital in managing the risks associated with stroke, particularly regarding hip fractures. By leveraging both traditional assessment methods and innovative digital solutions, healthcare providers can enhance their ability to identify at-risk patients and implement effective preventive strategies, thereby improving clinical outcomes for stroke survivors.

## 6 Clinical outcomes of hip fractures in stroke patients

### 6.1 Mortality and recurrence risk

Stroke and hip fracture outcomes are closely linked due to the high mortality and recurrence risks associated with both conditions. Patients who suffer a hip fracture after a stroke have markedly higher short-term mortality rates, especially within the first 30 days after surgery. Research indicates that the 30-day mortality rate for these patients can be as high as 14.8%. This high rate reflects the acute complications often associated with both conditions, such as pneumonia, myocardial infarction, and other systemic failures that can arise post-fracture (Chen et al., 2023; Weinstein et al., 2024).

Moreover, individuals with a prior stroke have a higher risk of recurrent fractures, especially hip fractures. This increased risk is due to impaired mobility and balance, as well as common comorbidities like osteoporosis in this population (Weinstein et al., 2024; Lamo-Espinosa et al., 2023). The risk of a second hip fracture can be particularly pronounced in patients who had good pre-fracture mobility, as they may return to activities that predispose them to further falls and fractures.

In summary, stroke combined with hip fracture worsens patient outcomes by increasing mortality and recurrence risks. Therefore, comprehensive post-stroke rehabilitation and preventive strategies are essential to reduce subsequent fractures. Addressing physical, environmental, and medical factors is critical to improving survival and quality of life in this vulnerable group.

### 6.2 Complications and functional recovery

Postoperative complications after stroke significantly affect recovery, especially in patients with hip fractures. These patients are highly prone to complications such as pneumonia and deep vein thrombosis (DVT), which can greatly hinder rehabilitation and extend hospital stays. Pneumonia is a common complication. Studies show it occurs in about 40% of patients receiving endovascular therapy for acute ischemic stroke, emphasizing the need for vigilant monitoring and prevention (Pilgram-Pastor et al., 2021). Additionally, the incidence of DVT in stroke patients can reach as high as 14.39%. Risk factors include prolonged bed rest and pre-existing conditions such as hypertension and diabetes (He et al., 2023). These complications not only delay functional recovery but also increase morbidity and mortality rates among stroke survivors.

Moreover, cognitive impairments and nutritional deficiencies further complicate the recovery process. Cognitive dysfunction affects a significant proportion of stroke patients and can exacerbate functional limitations, thereby hindering rehabilitation efforts. The prevalence of post-stroke cognitive impairment can be as high as 20% within the first 6 months following a stroke, often correlating with poorer functional outcomes (Schwarzbach and Grau, 2020). Additionally, malnutrition is a prevalent issue among stroke patients. Low serum albumin levels are associated with increased complications and poorer recovery trajectories (Sim et al., 2021). Nutritional support, such as tailored dietary plans and supplementation,is critical in this population, as it has been shown to facilitate better recovery outcomes and improve overall health status.

## 7 The reciprocal pathological burden between stroke and hip fractures

Hip fractures significantly exacerbate the pathological burden of stroke patients. They lead to increased pain, limited mobility, and prolonged hospitalization. The pain associated with hip fractures can be debilitating and cause a range of complications, such as infections, deep vein thrombosis, and pressure ulcers, which worsen the already fragile health status of stroke survivors. Studies indicate that the incidence of hip fractures in stroke patients is notably higher than in the general population, with a reported adjusted hazard ratio of 2.42 for hip fractures among stroke survivors compared to matched controls (Lee D. et al., 2024). This increased vulnerability results from many stroke patients experiencing impaired mobility and balance, which heighten their risk of falls and subsequent fractures. Consequently, the occurrence of a hip fracture not only intensifies the physical suffering of these patients but also exacerbates the functional limitations stemming from their stroke, further diminishing their quality of life. Moreover, the extended length of hospital stays associated with hip fractures adds to the healthcare burden and can lead to increased healthcare costs and resource utilization (Wahlsten et al., 2021; Fluck et al., 2023).

To transition from the physical impacts to the inflammatory and neurological aspects, it is important to consider the role of inflammation in recovery. In addition to the direct consequences of pain and immobility, the inflammatory response triggered by a hip fracture can adversely affect neurological recovery in stroke patients. Research has shown that the inflammatory mediators released during the fracture healing process can interfere with the brain’s ability to recover from ischemic injury, potentially leading to poorer functional outcomes (Hjelholt et al., 2022). The presence of a hip fracture may also complicate rehabilitation efforts, as the need for surgical intervention and the subsequent recovery period can delay or hinder the rehabilitation process for stroke patients, further entrenching their disability.

The psychological impact of experiencing a hip fracture after a stroke cannot be overlooked. The fear of falling and sustaining additional injuries can lead to increased anxiety and depression, which are already prevalent in stroke survivors. This psychological burden can deter patients from engaging in specific rehabilitation activities such as physical therapy and mobility training, thus perpetuating a cycle of decline in both physical and mental health (Fluck et al., 2023; Shen et al., 2022).

Put simply, including more pain, limited function, longer hospital stays, and worse neurological recovery. Addressing these challenges requires a comprehensive approach that includes effective pain management, early mobilization strategies, and targeted rehabilitation interventions to minimize the impact of hip fractures on stroke survivors. The integration of multidisciplinary care teams may be essential in optimizing outcomes for this vulnerable population, ensuring that both the physical and psychological aspects of recovery are adequately addressed.

## 8 Establishment and application of risk assessment and prediction models

### 8.1 Application of FRAX score in stroke patients

The Fracture Risk Assessment Tool (FRAX) has emerged as a pivotal instrument in predicting the risk of hip fractures among various populations, including stroke patients. Notably, a study showed that the FRAX score without bone mineral density (BMD) data effectively predicts hip fracture risk in stroke survivors. In a cohort of postmenopausal women, those with severe disability after stroke had a significantly higher risk of hip fractures, with a hazard ratio of 2.1 compared to those with favorable recovery. This association remained significant even after accounting for mortality. These findings underscore the importance of utilizing the FRAX score in clinical settings to identify individuals at high risk for hip fractures after stroke (Northuis et al., 2020). Incorporating FRAX into the standard care pathways for stroke survivors could facilitate early intervention strategies, such as osteoporosis screening and preventive treatment. These strategies are crucial given the high incidence of hip fractures in this population. By identifying those at greatest risk, clinicians can implement targeted preventive measures that directly reduce the burden of hip fractures and improve overall patient outcomes during the post-stroke recovery phase. This highlights the utility of the FRAX score not only as a predictive tool but also as a means to guide clinical decision-making in the management of bone health among stroke survivors.

### 8.2 Novel risk prediction models and tools

Developing novel risk prediction models and tools has become essential for managing patients at risk of falls and complications like hip fractures, especially after a stroke. Recent studies have highlighted the effectiveness of combining various clinical parameters—including the frequency of falls, the National Institutes of Health Stroke Scale (NIHSS) scores, and cognitive assessments like the Short Walk and Walk Test (SWWT)—to create robust predictive models. These models have demonstrated high accuracy in identifying individuals at elevated risk for adverse outcomes following a stroke, thereby facilitating timely and appropriate interventions. For instance, one study showed that incorporating these factors into a nomogram significantly improved risk stratification precision, enabling healthcare providers to tailor preventive strategies more effectively (Ha et al., 2021). Meanwhile, the application of nomograms and web-based calculators has enhanced the accessibility and usability of these predictive tools in clinical settings, enabling practitioners to make informed decisions rapidly. Such innovations not only streamline the risk assessment process but also empower healthcare professionals to implement personalized management plans that can mitigate the risks associated with falls and fractures in vulnerable populations, particularly stroke survivors. The ongoing refinement of these models, supported by real-world data and validation studies, underscores their potential to transform clinical practice and improve patient outcomes in the context of stroke-related complications (Lin et al., 2023; Yang et al., 2025).

## 9 Prevention strategies for hip fractures in stroke patients

### 9.1 Osteoporosis screening and pharmacological treatment

Osteoporosis screening and subsequent pharmacological treatment are critical components of managing bone health, particularly in post-stroke patients who are at an elevated risk for fragility fractures. Despite the known increased risk of osteoporosis in this population, current guidelines lack specific recommendations for routine screening, highlighting a significant gap in clinical practice. A scoping review has shown that less than 10% of post-stroke patients undergoes osteoporosis screening, underscoring the urgent need for standardized protocols to identify individuals at risk (Knight et al., 2025). Early detection through screening can facilitate timely interventions, including the initiation of anti-osteoporosis medications, which have been shown to effectively reduce fracture risk. Notably, bisphosphonates remain a first-line treatment for many patients, while newer agents such as denosumab and teriparatide also offer promising results in enhancing bone density and reducing fracture incidence (Malaise et al., 2020; Tay et al., 2022).

Moreover, recent studies have suggested that statins, commonly used for managing cholesterol levels, may also confer protective effects against fractures. This adds complexity to the management of osteoporosis in patients with comorbid conditions, affecting aspects such as diagnosis, treatment choices, and potential drug interactions (Bao et al., 2023). The interplay between osteoporosis and other health conditions, particularly in the elderly and those recovering from strokes, necessitates a multifaceted approach to treatment. This approach should include not only pharmacological interventions but also lifestyle modifications, such as diet and exercise (Jiang et al., 2023).

The lack of awareness and adherence to osteoporosis treatment regimens among healthcare providers and patients alike contributes to the underdiagnosis and undertreatment of osteoporosis. For instance, a significant proportion of patients who are eligible for osteoporosis medications do not receive them, often due to misconceptions about the necessity and safety of these treatments (Tay et al., 2022). This highlights the need for enhanced education and communication strategies to inform both patients and healthcare providers about the importance of osteoporosis screening and treatment.

In conclusion, the integration of routine osteoporosis screening into the clinical management of post-stroke patients, coupled with timely pharmacological treatment, is essential for reducing the risk of fractures and improving overall patient outcomes. As the population ages and the prevalence of osteoporosis increases, it is imperative to establish clear guidelines and enhance awareness among healthcare professionals to ensure that at-risk patients receive appropriate care (Malyavko et al., 2025).

### 9.2 Exercise rehabilitation and fall prevention

Exercise rehabilitation is a crucial component in the management of stroke patients, particularly in minimizing the risk of falls, which are a significant concern in this population. Research indicates that tailored rehabilitation programs focusing on balance and strength training can effectively reduce the incidence of falls among stroke survivors. A systematic review showed that structured exercise, including balance training, significantly improves stability and physical function, thus reducing fall risk (Alfrey et al., 2024). It should also be noted that early rehabilitation post-stroke has been shown to mitigate the onset of disability, with studies indicating that patients who engage in early mobilization and strength training exhibit better recovery outcomes and a reduced fall rate (Lin et al., 2021). This emphasizes the necessity of initiating rehabilitation promptly after a stroke to enhance recovery and prevent falls.

Besides physical interventions, environmental factors also play a critical role in fall prevention strategies for stroke patients. Adaptations in the living environment, such as removing trip hazards, improving lighting, and installing grab bars, can significantly enhance safety and mobility for stroke survivors. The use of assistive devices, including walkers and canes, is also recommended to provide additional support during ambulation (Kim et al., 2022). These modifications, when combined with exercise rehabilitation, create a comprehensive approach to fall prevention that addresses both physical capabilities and environmental safety.

In conclusion, a multifaceted approach that combines exercise rehabilitation, environmental modifications, and the use of technology is essential for effective fall prevention in stroke patients. By addressing both the physical and environmental factors contributing to fall risk, healthcare providers can significantly improve the safety and quality of life for stroke survivors. Continuous research and innovation in rehabilitation practices will further enhance the effectiveness of these strategies, ensuring that stroke patients receive the comprehensive care they need to thrive post-stroke.

### 9.3 Lifestyle interventions

Lifestyle interventions play a crucial role in promoting bone health, especially in populations at risk for conditions such as hip fractures following events like stroke. Smoking cessation, adequate nutrition, and physical activity are key components of these interventions. Smoking has been consistently linked to decreased bone density and impaired skeletal health, including increased bone resorption and delayed bone healing. Research indicates that smoking cessation can lead to improved bone mineral density and a reduced risk of fractures, highlighting the importance of addressing this modifiable risk factor in clinical management (Sandvik, 2020; Davis and Lorig, 2024). Furthermore, nutritional supplementation, particularly with protein and vitamin D, has been shown to be beneficial for bone health. Protein is essential for maintaining muscle mass, which supports bone structure and helps prevent falls, while vitamin D is vital for calcium absorption and bone remodeling (Sherzai et al., 2022; Ard et al., 2024). A study demonstrated that adequate dietary protein intake is associated with higher bone mineral density in older adults, suggesting that dietary interventions should emphasize protein-rich foods, particularly for those at risk of osteoporosis and fractures (Rivers, 2022). Vitamin D supplementation, especially in populations with limited sun exposure, can significantly enhance bone health and reduce fracture risk (Salzberg, 2022).

Physical activity is another cornerstone of lifestyle interventions. Regular weight-bearing exercises have been shown to improve bone density and overall musculoskeletal health. Engaging in activities such as walking, dancing, or resistance training can stimulate bone formation and increase muscle strength, which is vital for preventing falls and subsequent fractures (Rutters et al., 2024). Moreover, the combination of dietary improvements and physical activity has a synergistic effect on bone health, providing a multifaceted approach to prevention. For instance, a study indicated that individuals who combined a balanced diet rich in calcium and vitamin D with regular physical activity had significantly better bone health outcomes than those who did not (Chang et al., 2023).

In essence, lifestyle interventions that include smoking cessation, adequate nutritional intake (particularly protein and vitamin D), and regular physical activity are essential strategies for enhancing bone health and preventing hip fractures, especially in vulnerable populations such as stroke survivors. These interventions should be integrated into clinical practice to promote better health outcomes and improve quality of life for individuals at risk of skeletal-related complications (Table 1).

**TABLE 1:** Prevention strategies for hip fractures in stroke patients: a “4S framework”.

Framework component	Key actions	Timing of intervention	Clinical pearls
Screen	- FRAX assessment without BMD - NIHSS and berg balance scale - Fall risk evaluation	- Within 1 month post-stroke - Repeat annually or after clinical change	FRAX effectively predicts hip fracture risk in stroke patients even without BMD data. Prioritize screening in those with NIHSS >10 or BBS <45
Strengthen	- Task-oriented balance training - Lower extremity strengthening - Gait and proprioception exercises	- Begin in acute rehabilitation phase - Continue through chronic phase	Early mobilization within 48–72 h post-stroke reduces fall risk. Dual-task training (motor + cognitive) is particularly effective
Supplement	- Calcium (1,000–1,200 mg/day) - Vitamin D (800–1000 IU/day) - Consider bisphosphonates if T-score ≤ −2.5	- Initiate immediately post-stroke - Continue long-term	Vitamin D deficiency affects >50% of stroke survivors. Start supplementation without waiting for BMD results in high-risk patients
Modify	- Home safety assessment - Remove trip hazards - Install grab bars, improve lighting - Appropriate assistive devices	- Before discharge from rehabilitation - Reassess after any fall	Home modifications reduce falls by 30%–40%. Prescribe walking aids based on specific deficits (e.g., quad cane for severe hemiparesis)

## 10 Surgical and perioperative management of hip fractures in stroke patients

### 10.1 Timing of surgery and indications assessment

The timing of surgical intervention for patients who have suffered a hip fracture following a stroke is a critical consideration that must be tailored to individual patient circumstances, including the type of stroke, its severity, and the functional status of the patient. A personalized surgical plan is essential, as the optimal timing for surgery can significantly influence postoperative outcomes. Studies have shown that delays in surgical intervention can lead to increased morbidity and mortality, particularly in older adults with hip fractures. For instance, a retrospective cohort study indicated that patients undergoing hip fracture surgery after a delay of more than 48 h had a higher risk of mortality and complications, such as myocardial infarction and stroke, compared to those who received timely surgical care (Matthews et al., 2021; Jian et al., 2021). Therefore, it is imperative to assess the patient’s overall health status, including comorbidities and functional capacity, to determine the most appropriate timing for surgery.

More importantly, the collaboration of a multidisciplinary team is vital in optimizing the timing of surgery. This team typically includes orthopedic surgeons, geriatricians, anesthesiologists, and rehabilitation specialists, all of whom contribute to a comprehensive evaluation of the patient’s condition. For example, in patients with acute stroke, the presence of neurological deficits and the type of stroke (ischemic vs. hemorrhagic) can dictate the urgency of surgical intervention. A study highlighted that patients with ischemic strokes may benefit from earlier surgical intervention, as this can mitigate the risk of further complications (Nikatov et al., 2024). The integration of various specialties ensures that all aspects of the patient’s health are considered, leading to a more informed decision regarding the timing of surgery.

Essentially, the assessment of surgical timing and indications for hip fracture repair in patients with a history of stroke requires a nuanced approach that considers individual patient factors and the collaborative efforts of a multidisciplinary team. Timely intervention is crucial to reduce the risk of adverse outcomes, and ongoing evaluation of the patient’s condition is necessary to adapt the surgical plan as needed. This comprehensive strategy not only enhances patient safety but also improves overall surgical outcomes in this vulnerable population.

### 10.2 Perioperative complication prevention and control

The management of perioperative complications in patients undergoing hip surgery, particularly those with a history of stroke, is critical for improving outcomes and minimizing morbidity. Effective blood pressure management is paramount, as fluctuations can lead to adverse cardiovascular events during the perioperative period. Studies have shown that maintaining normotension can significantly reduce the risk of complications such as myocardial infarction and stroke recurrence (Li, 2024; Mitrică et al., 2024). Furthermore, proactive measures such as the use of beta-blockers and careful fluid management can help stabilize hemodynamics, particularly in patients with pre-existing cardiovascular conditions (Takagi et al., 2021). The prevention of pulmonary infections is another crucial aspect of perioperative care. Patients with compromised respiratory function, such as those with a history of stroke, are at an increased risk for postoperative pneumonia. Implementing strategies like incentive spirometry, early mobilization, and the use of prophylactic antibiotics can mitigate this risk (Nobuhara et al., 2022; Yamguchi et al., 2024). Additionally, the incorporation of preoperative oral care has been shown to decrease the incidence of surgical site infections, further enhancing recovery (Yilmaz Eker and Yilmaz, 2023).

Early nutritional support and functional rehabilitation are essential components of postoperative care that can significantly influence recovery trajectories. Research indicates that early nutritional intervention can promote wound healing and reduce the incidence of complications such as infections and delayed recovery (Liu et al., 2022). Moreover, engaging patients in functional exercises soon after surgery has been linked to improved mobility and reduced hospital stays, which is particularly beneficial for older adults who may face prolonged recovery periods (Zhang et al., 2024).

Ultimately, the prevention and management of perioperative complications in patients with hip fractures, especially those with a history of stroke, require a comprehensive strategy that encompasses meticulous blood pressure control, infection prevention, and early rehabilitation efforts. By implementing these measures, healthcare providers can significantly enhance patient outcomes, reduce hospital stays, and ultimately improve the quality of life for these vulnerable populations (Table 2).

**TABLE 2:** Clinical pearls for perioperative management of hip fractures in stroke patients.

Phase	Key considerations	Actionable recommendations
Preoperative (0–48 h)	- Optimize medical comorbidities - Assess stroke severity and type - Evaluate bleeding risk	Pearl 1: Delay surgery >48 h only if medically necessary—each 24-h delay increases 30-day mortality by 5% Pearl 2: For patients on antiplatelets, consult cardiology/neurology before discontinuation—risk of stroke recurrence often outweighs bleeding risk
Intraoperative	- Hemodynamic stability - Anesthesia type selection - Stroke recurrence prevention	Pearl 3: Maintain mean arterial pressure within 20% of baseline—hypotension is the most common trigger for perioperative stroke recurrence Pearl 4: Regional anesthesia preferred when feasible—reduces delirium, pneumonia, and thromboembolic events
Postoperative (0–7 days)	- Early mobilization - Thromboembolism prophylaxis - Delirium prevention	Pearl 5: Mobilize within 24 h post-surgery—bed rest for >48 h increases pneumonia risk by 40% Pearl 6: Use multimodal analgesia to avoid oversedation—opioids increase fall risk and delirium
Rehabilitation (7 days+)	- Stroke-specific rehabilitation - Fall prevention education - Bone health optimization	Pearl 7: Integrate stroke and fracture rehabilitation—patients need both neurological recovery and fracture healing Pearl 8: Screen for osteoporosis immediately post-fracture—only 10% of patients currently receive appropriate treatment

### 10.3 Postoperative rehabilitation and long-term follow-up

Postoperative rehabilitation and long-term follow-up are critical components in the management of patients who have suffered hip fractures, particularly in the context of stroke recovery. The development of targeted rehabilitation plans is essential to enhance patients’ independence in daily living activities. Research indicates that a comprehensive rehabilitation approach, which includes physical therapy and occupational therapy, significantly improves functional outcomes in older adults post-hip fracture. For instance, a study highlighted that tailored rehabilitation programs can lead to better mobility and self-care abilities, thereby reducing the risk of complications such as further fractures or falls (Rudy and Saller, 2023). Furthermore, the integration of multidisciplinary teams, including physiatrists, physical therapists, and nurses, ensures that rehabilitation is holistic and addresses the multifaceted needs of the patient. This collaborative approach not only focuses on physical recovery but also considers psychological support, which is vital for enhancing the overall quality of life post-fracture.

Long-term monitoring of patients who have experienced both a stroke and a hip fracture is equally important. Continuous assessment of the risk of recurrent fractures and the potential for stroke recurrence should be a priority in follow-up care. Studies have shown that stroke survivors are at a heightened risk for subsequent hip fractures, with the incidence being significantly higher compared to non-stroke patients (Zhang et al., 2023). This necessitates regular evaluations of bone mineral density and functional assessments to adjust rehabilitation strategies accordingly. For example, the use of tools such as the Functional Independence Measure (FIM) can help in tracking the progress of rehabilitation and identifying areas needing further intervention (Mikayama et al., 2024). Additionally, it is crucial to monitor the psychological wellbeing of these patients, as depression and anxiety can adversely affect rehabilitation outcomes. Therefore, implementing a structured follow-up program that includes both physical and mental health assessments can significantly enhance recovery trajectories for patients recovering from hip fractures following a stroke.

Effective postoperative rehabilitation combined with diligent long-term follow-up is paramount for optimizing recovery in patients with hip fractures, particularly those with a history of stroke. By focusing on individualized rehabilitation plans and continuous monitoring of both physical and psychological health, healthcare providers can significantly improve the quality of life and functional independence for these vulnerable populations.

## 11 Limitations of current evidence and future research priorities

Despite the growing body of evidence elucidating the interplay between stroke and hip fractures, several limitations within the existing literature warrant consideration. First, the majority of available studies are observational in nature, which inherently limits the ability to establish causal relationships and may be subject to confounding biases. Second, there is considerable heterogeneity across studies in terms of population characteristics (e.g., stroke severity, type, and chronicity), fracture definitions, and outcome assessment tools, which complicates the synthesis and generalizability of findings. Third, while tools such as the FRAX score have shown utility in predicting hip fracture risk among stroke survivors, their calibration remains suboptimal for this specific population, as they do not currently incorporate stroke-related variables such as hemiparesis, spasticity, or fall frequency. Additionally, evidence regarding the optimal timing for initiating osteoporosis pharmacotherapy post-stroke is scarce, and clinical guidelines lack consensus on when and how to screen for bone health in this high-risk group.

To address these gaps, future research should prioritize the following key areas: (1) the development and validation of stroke-specific fracture risk prediction models that integrate neurological deficits, fall risk, and bone metabolism markers; (2) prospective cohort studies or randomized controlled trials to determine the optimal timing and efficacy of anti-osteoporosis interventions in the early post-stroke phase; (3) mechanistic studies to clarify the temporal dynamics of bone loss and the role of neuroinflammatory and endocrine pathways; and (4) implementation science research to evaluate the feasibility and impact of integrated care models combining fall prevention, bone health management, and stroke rehabilitation. Addressing these priorities will be essential to advance personalized prevention strategies and improve long-term outcomes for stroke survivors at risk of hip fractures.

## 12 Conclusion

Stroke and hip fractures represent a critical intersection in post-stroke care, characterized by elevated risks of mortality and recurrent fracture. This review underscores that fracture risk in stroke survivors is primarily driven by falls and secondary osteoporosis, necessitating integrated risk assessment tools such as FRAX alongside comprehensive clinical evaluation. Preventive strategies must center on osteoporosis screening and individualized rehabilitation, delivered through multidisciplinary collaboration among neurologists, orthopedists, and rehabilitation specialists.

Future research should prioritize elucidating the shared pathophysiological mechanisms linking stroke and bone fragility, and developing targeted interventions to mitigate fracture risk. Advancing understanding in this domain will be essential to reducing hip fracture incidence and improving long-term outcomes and quality of life for stroke survivors.

## References

[B1] Alfrey K. L. Gardner B. Judd J. Askew C. D. Vandelanotte C. Rebar A. L. (2024). Physical activity behaviour and motivation during and following pulmonary and cardiac rehabilitation: a repeated measures study. Behav. Sci. (Basel). 14 (10), 965. 10.3390/bs14100965 39457837 PMC11504991

[B2] Ard J. D. Lewis K. H. Moore J. B. (2024). Lifestyle interventions for obesity-reply. JAMA 332 (17), 1488–1489. 10.1001/jama.2024.17536 39382859

[B3] Austin T. R. Nethander M. Fink H. A. Törnqvist A. E. Jalal D. I. Buzkova P. (2024). A plasma protein-based risk score to predict hip fractures. Nat. Aging 4 (8), 1064–1075. 10.1038/s43587-024-00639-7 38802582 PMC11333168

[B4] Bao C. Wu T. Zhu S. Wang X. Zhang Y. Wang X. (2023). Regulation of cholesterol homeostasis in osteoporosis mechanisms and therapeutics. Clin. Sci. (Lond). 137 (15), 1131–1143. 10.1042/CS20220752 37553962

[B5] Borges F. K. Guerra-Farfan E. Bhandari M. Patel A. Slobogean G. Feibel R. J. (2024). Myocardial injury in patients with hip fracture: a HIP ATTACK randomized trial substudy. J. Bone Jt. Surg. Am. 106 (24), 2303–2312. 10.2106/JBJS.23.01459 39052767

[B6] Cai Y. Gu H. Li L. Liu X. Bai Y. Shen L. (2024). New TIPARP inhibitor rescues mitochondrial function and brain injury in ischemic stroke. Pharmacol. Res. 210, 107508. 10.1016/j.phrs.2024.107508 39547463

[B7] Carcreff L. Armand S. Bonnefoy-Mazure A. De Coulon G. Newman C. J. (2022). New tools for movement assessment in children with motor disabilities. Rev. Med. Suisse 18 (770), 336–339. 10.53738/REVMED.2022.18.770.336 35224909

[B8] Chang M. W. Wegener D. T. Tan A. Schaffir J. Worly B. Strafford K. (2023). Pilot lifestyle intervention effect on lifestyle behaviors, psychosocial factors, and affect. J. Pediatr. Perinatol. Child. Health 7 (2), 74–82. 10.26502/jppch.74050147 38576861 PMC10994102

[B9] Chen P. Zhang W. Qi J. Yang B. Fan Z. Chen Y. (2023). The incidence and risk factors of perioperative recurrent stroke in elderly patients with previous ischemic stroke receiving hip fracture surgery. BMC Musculoskelet. Disord. 25 (1), 636. 10.1186/s12891-024-07753-y 39127635 PMC11316288

[B10] Chen C. He K. Morgenstern L. B. Shi X. Shafie-Khorassani F. Lisabeth L. D. (2025). Trends and ethnic differences in stroke recurrence and mortality in a biethnic population, 2000-2019: a novel application of an illness-death model. Ann. Epidemiol. 85, 51–58.e5. 10.1016/j.annepidem.2023.04.003 37054958

[B11] Davis C. L. Lorig K. (2024). Lifestyle interventions for obesity. JAMA 332 (17), 1488. 10.1001/jama.2024.17533 39382884

[B12] Delmas S. Tiwari A. Tseng H. Y. Poisson S. N. Diehl M. Lodha N. (2025). Amplified intraindividual variability in motor performance in stroke survivors: links to cognitive and clinical outcomes. Brain Behav. 15 (2), e70365. 10.1002/brb3.70365 39972991 PMC11839749

[B13] Fereidoonnezhad B. Dwivedi A. Johnson S. McCarthy R. McGarry P. (2021). Blood clot fracture properties are dependent on red blood cell and fibrin content. Acta Biomater. 127, 213–228. 10.1016/j.actbio.2021.03.052 33812070

[B14] Fluck D. Lisk R. Yeong K. Mahmood R. Robin J. Fry C. H. (2023). Sex differences in clinical outcomes amongst 1105 patients admitted with hip fractures. Intern Emerg. Med. 18 (5), 1561–1568. 10.1007/s11739-023-03264-1 37101056

[B15] Fu Y. Wang C. Zhang L. Ji D. Xiang A. Qi J. (2024). The effectiveness of theta burst stimulation for motor recovery after stroke: a systematic review. Eur. J. Med. Res. 29 (1), 568. 10.1186/s40001-024-02170-2 39609900 PMC11605871

[B16] Gao Z. Chen Z. Xiong Z. Liu X. (2022). Ferroptosis - a new target of osteoporosis. Exp. Gerontol. 165, 111836. 10.1016/j.exger.2022.111836 35598699

[B17] Ha A. S. Pak J. Haas C. R. Miles C. Weiner D. M. Anderson C. B. (2021). A novel risk prediction model to triage difficult urethral catheterizations. Urology 157, 35–40. 10.1016/j.urology.2021.05.059 34153365

[B18] He J. Wang F. Xiong L. L. Sun H. Y. Li W. (2023). The occurrence and risk factors of stroke complicated by venous thromboembolism. Sichuan Da Xue Xue Bao Yi Xue Ban. 54 (3), 638–641. 10.12182/20230560104 37248597 PMC10475418

[B19] Hjelholt T. J. Johnsen S. P. Brynningsen P. K. Pedersen A. B. (2022). The interaction effect between previous stroke and hip fracture on postoperative mortality: a nationwide cohort study. Clin. Epidemiol. 14, 543–553. 10.2147/CLEP.S361507 35509521 PMC9058007

[B20] Hsieh C. Y. Sung S. F. Huang H. K. (2020). Drug treatment strategies for osteoporosis in stroke patients. Expert Opin. Pharmacother. 21 (7), 811–821. 10.1080/14656566.2020.1736556 32151211

[B21] Ibsen C. Schiøttz-Christensen B. Vinther Nielsen C. Hørder M. Schmidt A. M. Maribo T. (2022). Assessment of functioning and disability in patients with low back pain - the low back pain assessment tool. Part 1: development. Disabil. Rehabil. 44 (17), 4841–4852. 10.1080/09638288.2021.1913648 33945363

[B22] Jian M. Y. Liang F. Liu H. Y. Han R. Q. (2021). Perioperative massive cerebral stroke in thoracic patients: report of three cases. World J. Clin. Cases 9 (13), 3170–3176. 10.12998/wjcc.v9.i13.3170 33969105 PMC8080756

[B23] Jiang Y. Zhu Y. Zhang B. Feng D. (2023). Characteristics of subsequent contralateral proximal femoral fracture: more convenient access is needed to treat osteoporosis. J. Orthop. Surg. Res. 18 (1), 126. 10.1186/s13018-023-03621-y 36810116 PMC9945589

[B24] Kim J. Song J. Kim D. Park J. (2022). The development of ICT-based exercise rehabilitation service contents for patients with musculoskeletal disorders and stroke. Int. J. Environ. Res. Public Health 19 (9), 5022. 10.3390/ijerph19095022 35564415 PMC9106069

[B25] Knight S. Swance R. Bateman E. A. Clemens K. K. Papaioannou A. Fleet J. L. (2025). Post-stroke osteoporosis screening: a scoping review. Cerebrovasc. Dis. 54, 1–11. 10.1159/000542924 39626648

[B26] Kumar A. Shahvej S. K. Yadav P. Modi U. Yadav A. K. Solanki R. (2025). Clinical applications of targeted nanomaterials. Pharmaceutics 17 (3), 379. 10.3390/pharmaceutics17030379 40143042 PMC11944548

[B27] Lamo-Espinosa J. M. Mariscal G. Gómez-Álvarez J. San-Julián M. (2023). Incidence and risk factors for stroke after hip fracture: a meta-analysis. Sci. Rep. 13 (1), 17618. 10.1038/s41598-023-44917-7 37848510 PMC10582073

[B28] Lee J. Kim H. Moon J. Shin J. Jeong H. Kim Y. (2022). Temporal trend of first-ever ischaemic stroke incidence from 2010 to 2019 in South Korea: a nationwide retrospective cohort study. BMJ Open 12 (8), e059956. 10.1136/bmjopen-2021-059956 36002224 PMC9413172

[B29] Lee J. H. Han K. D. Cheon D. Y. Lee M. (2024). Association between changes in smoking habits and incident fracture after acute ischemic stroke. J. Am. Heart Assoc. 13 (11), e034779. 10.1161/JAHA.124.034779 38804231 PMC11255617

[B30] Lee D. Cho I. Y. Chang W. H. Yoo J. E. Choi H. L. Park J. (2024). Fracture risk among stroke survivors according to poststroke disability status and stroke type. Stroke 55 (6), 1498–1506. 10.1161/STROKEAHA.123.044953 38686561

[B31] Li H. (2024). Pay attention to the identification and management of perioperative stroke. Zhonghua Wai Ke Za Zhi 62 (7), 619–623. 10.3760/cma.j.cn112139-20240313-00123 38808427

[B32] Li J. Qiu H. Zhang X. Jin J. Zhao Y. Yan J. (2022). Validation of a disability assessment tool based on the international classification of functioning, disability, and health in the Chinese context. Front. Rehabil. Sci. 3, 855502. 10.3389/fresc.2022.855502 36189056 PMC9397936

[B33] Lin R. C. Lin C. H. Weng S. M. Lin C. F. Wang W. Y. Tzeng W. C. (2021). Applying multiple strategies to increase the rate of early rehabilitation exercise adoption in patients with acute stroke. Hu Li Za Zhi 68 (1), 64–73. 10.6224/JN.202102_68(1).09 33521920

[B34] Lin Y. Wang Z. Liu S. (2023). Risk factors and novel predictive models for metastatic neuroblastoma in children. Eur. J. Surg. Oncol. 49 (12), 107110. 10.1016/j.ejso.2023.107110 37862722

[B35] Liu Y. Zheng Y. Reddy A. S. Gebrezgiabhier D. Davis E. Cockrum J. (2021). Analysis of human emboli and thrombectomy forces in large-vessel occlusion stroke. J. Neurosurg. 134 (3), 893–901. 10.3171/2019.12.JNS192187 32109875

[B36] Liu J. Chen L. Long C. Zhang X. Gao F. Duan X. (2022). Effect of a preoperative mobilization program on perioperative complications and function recovery in older adults with femoral neck fracture. Geriatr. Nurs. 44, 69–75. 10.1016/j.gerinurse.2022.01.003 35074539

[B37] Liu B. Ng C. Y. La P. B. D. Wong P. Ebeling P. R. Singhal S. (2024). Osteoporosis and fracture risk assessment in adults with ischaemic stroke. Osteoporos. Int. 35 (7), 1243–1247. 10.1007/s00198-024-07099-0 38703219 PMC11211165

[B38] Lu F. Li P. Zeng F. (2024). Exploration of a somatosensory interactive assessment tool for children with intellectual disabilities. Psych. J. 13 (5), 804–812. 10.1002/pchj.759 38632076 PMC11444717

[B39] Malaise O. Detroz M. Leroy M. Leonori L. Seidel L. Malaise M. G. (2020). High detection rate of osteoporosis with screening of a general hospitalized population: a 6-year study in 6406 patients in a university hospital setting. BMC Musculoskelet. Disord. 21 (1), 90. 10.1186/s12891-020-3116-9 32041590 PMC7011267

[B40] Malyavko A. Agarwal A. R. Mikula J. D. Best M. J. Srikumaran U. (2025). Shoulder arthroplasty patients are underscreened for osteoporosis. J. Am. Acad. Orthop. Surg. 33 (7), 362–369. 10.5435/JAAOS-D-23-00408 39637424

[B41] Marquez-Romero J. M. Romo-Martínez J. Hernández-Curiel B. Ruiz-Franco A. Krishnamurthi R. Feigin V. (2024). Assessing the individual risk of stroke in caregivers of patients with stroke. Arq. Neuropsiquiatr. 82 (3), 1–5. 10.1055/s-0044-1779691 38467391 PMC10927366

[B42] Matthews C. R. Hartman T. Madison M. Villelli N. W. Namburi N. Colgate C. L. (2021). Preoperative stroke before cardiac surgery does not increase risk of postoperative stroke. Sci. Rep. 11 (1), 9025. 10.1038/s41598-021-88441-y 33907259 PMC8079406

[B43] Mikayama S. Kubo T. Tahara T. Nakamura M. Oku F. Kenmochi K. (2024). Prognostic equations and accuracy of a total score of functional Independence measure at discharge for different diseases in a convalescent rehabilitation ward. Cureus 16 (8), e66509. 10.7759/cureus.66509 39252717 PMC11382432

[B44] Mitrică M. Lorusso L. Badea A. A. Sîrbu C. A. Pleșa A. Stănescu A. M. A. (2024). The hidden heart: exploring cardiac damage post-stroke: a narrative review. Med. Kaunas. 60 (10), 1699. 10.3390/medicina60101699 39459486 PMC11509537

[B45] Nikatov K. Dyussembekov E. Kastey R. Zhukov Y. Yerniyazov N. Korabayev M. (2024). An impact of treatment initiation timing on stroke outcome: bridging the time gap. Med. Glas. (Zenica) 21 (1). 10.17392/1658-23 37950659

[B46] Nobuhara H. Matsugu Y. Soutome S. Hayashida S. Hasegawa T. Akashi M. (2022). Perioperative oral care can prevent surgical site infection after colorectal cancer surgery: a multicenter, retrospective study of 1,926 cases analyzed by propensity score matching. Surgery 172 (2), 530–536. 10.1016/j.surg.2022.02.015 35396104

[B47] Northuis C. A. Crandall C. J. Margolis K. L. Diem S. J. Ensrud K. E. Lakshminarayan K. (2020). Association between post-stroke disability and 5-year hip-fracture risk: the Women’s health initiative. J. Stroke Cerebrovasc. Dis. 29 (8), 104976. 10.1016/j.jstrokecerebrovasdis.2020.104976 32689623 PMC7394038

[B48] Oquita R. Cuello V. Uppati S. Mannuru S. Salinas D. Dobbs M. (2024). Moving toward elucidating alternative motor pathway structures post-stroke: the value of spinal cord neuroimaging. Front. Neurol. 15, 1282685. 10.3389/fneur.2024.1282685 38419695 PMC10899520

[B49] Orassi V. Duda G. N. Heiland M. Fischer H. Rendenbach C. Checa S. (2021). Biomechanical assessment of the validity of sheep as a preclinical model for testing mandibular fracture fixation devices. Front. Bioeng. Biotechnol. 9, 672176. 10.3389/fbioe.2021.672176 34026745 PMC8134672

[B50] Ozkan H. Ambler G. Banerjee G. Browning S. Leff A. P. Ward N. S. (2024). Prevalence, patterns, and predictors of patient-reported non-motor outcomes at 30 days after acute stroke: prospective observational hospital cohort study. Int. J. Stroke 19 (4), 442–451. 10.1177/17474930231215660 37950351 PMC10964387

[B51] Pilgram-Pastor S. M. Piechowiak E. I. Dobrocky T. Kaesmacher J. Den Hollander J. Gralla J. (2021). Stroke thrombectomy complication management. J. Neurointerv Surg. 13 (10), 912–917. 10.1136/neurintsurg-2021-017349 34158401 PMC8458081

[B52] Rivers D. (2022). Lifestyle interventions for cancer survivors. Nat. Rev. Cancer 22 (3), 130. 10.1038/s41568-021-00434-1 34893766

[B53] Rudy M. Saller T. (2023). Postoperative delirium in the recovery room. Anaesthesiologie 72 (7), 459–466. 10.1007/s00101-023-01281-5 37233791

[B54] Rutters F. den Braver N. R. Lakerveld J. Mackenbach J. D. van der Ploeg H. P. Griffin S. (2024). Lifestyle interventions for cardiometabolic health. Nat. Med. 30 (12), 3455–3467. 10.1038/s41591-024-03373-0 39604492

[B55] Salehi Omran S. Murthy S. B. Navi B. B. Merkler A. E. (2020). Long-term risk of hip fracture after ischemic stroke. Neurohospitalist 10 (2), 95–99. 10.1177/1941874419859755 32373271 PMC7191655

[B56] Salzberg L. (2022). Risk factors and lifestyle interventions. Prim. Care 49 (2), 201–212. 10.1016/j.pop.2021.11.001 35595477

[B57] Sandvik J. (2020). Surgery or lifestyle interventions? Tidsskr. Nor. Laegeforen 140 (16). 10.4045/tidsskr.20.0819 33172239

[B58] Schwarzbach C. J. Grau A. J. (2020). Complications after stroke: clinical challenges in stroke aftercare. Nervenarzt 91 (10), 920–925. 10.1007/s00115-020-00988-9 32914296

[B59] Shen S. Chu T. Wang J. Zhao H. Tang J. Xu L. (2025). Progress in the application of motor imagery therapy in upper limb motor function rehabilitation of stroke patients with hemiplegia. Front. Neurol. 16, 1454499. 10.3389/fneur.2025.1454499 39974369 PMC11835663

[B60] Shen Y. L. Zhang Z. Q. Zhu L. J. Liu J. H. (2022). Timing theory continuous nursing, resistance training: rehabilitation and mental health of caregivers and stroke patients with traumatic fractures. World J. Clin. Cases 10 (5), 1508–1516. 10.12998/wjcc.v10.i5.1508 35211588 PMC8855261

[B61] Sherzai D. Sherzai A. Sherzai A. (2022). Lifestyle intervention and alzheimer disease. J. Fam. Pract. 71 (Suppl. 1 Lifestyle), eS83–eS89. 10.12788/jfp.0286 35389852

[B62] Shi Y. (2021). The investigation of energy metabolism in osteoblasts and osteoclasts. Hua Xi Kou Qiang Yi Xue Za Zhi 39 (5), 501–509. 10.7518/hxkq.2021.05.002 34636196 PMC8548224

[B63] Shi X. Zhao J. Xu S. Ren M. Wu Y. Chen X. (2023). Clinical research progress of the post-stroke upper limb motor function improvement via transcutaneous auricular vagus nerve stimulation. Neural Plast. 2023, 9532713. 10.1155/2023/9532713 37789954 PMC10545466

[B64] Sim S. D. Sim Y. E. Tay K. Howe T. S. Png M. A. Chang C. C. P. (2021). Preoperative hypoalbuminemia: poor functional outcomes and quality of life after hip fracture surgery. Bone 143, 115567. 10.1016/j.bone.2020.115567 32745690

[B65] Singer M. (2021). Osteoporosis: common side effect. Clin. J. Oncol. Nurs. 25 (6), 13–15. 10.1188/21.CJON.S2.13-15 34800118

[B66] Sun K. Wang Y. Du J. Wang Y. Liu B. Li X. (2023). Exploring the mechanism of traditional Chinese medicine in regulating gut-derived 5-HT for osteoporosis treatment. Front. Endocrinol. (Lausanne) 14, 1234683. 10.3389/fendo.2023.1234683 37916145 PMC10616894

[B67] Takagi Y. Koyama M. Miyagawa Y. Kitazawa M. Kimura K. Soejima Y. (2021). A case of sigmoidectomy for sigmoid Colon cancer with severe pulmonary arterial hypertension associated with mixed tissue connected disease: a case report. Int. J. Surg. Case Rep. 83, 105906. 10.1016/j.ijscr.2021.105906 34023548 PMC8164025

[B68] Talbot S. Denton H. Dodds M. K. Lynch D. (2022). Changing trends in hip fracture epidemiology in the Republic of Ireland: a follow-up study. Arch. Osteoporos. 17 (1), 79. 10.1007/s11657-022-01112-x 35575820

[B69] Tan S. Liu Y. Li F. Han M. Ye M. (2020). The impact of various antiplatelet strategies on the incidence of gouty arthritis in hospitalized patients with acute cerebral infarction. Clin. Neurol. Neurosurg. 242, 108326. 10.1016/j.clineuro.2024.108326 38772278

[B70] Tay C. L. Ng W. L. Beh H. C. Lim W. C. Hussin N. (2022). Screening and management of osteoporosis: a survey of knowledge, attitude and practice among primary care physicians in Malaysia. Arch. Osteoporos. 17 (1), 72. 10.1007/s11657-022-01111-y 35474021 PMC9041673

[B71] Trojniak J. Sendera A. Banaś-Ząbczyk A. Kopańska M. (2024). The MicroRNAs in the pathophysiology of osteoporosis. Int. J. Mol. Sci. 25 (11), 6240. 10.3390/ijms25116240 38892426 PMC11172499

[B72] Wahlsten L. R. Zareini B. Smedegaard L. Gislason G. H. Palm H. Brorson S. (2021). A medical history of arterial thrombosis is a strong predictor of post-operative myocardial infarction and stroke in patients with hip fractures-a nationwide cohort study. Age Ageing 50 (4), 1252–1260. 10.1093/ageing/afaa279 33507243

[B73] Wan X. Zhu Z. Xu F. Li Q. (2025). Association between gait characteristics during obstacle crossing and fall risk in stroke patients: a prospective cohort study. Gait Posture 120, 9–16. 10.1016/j.gaitpost.2025.03.022 40179655

[B74] Wang H. P. Sung S. F. Yang H. Y. Huang W. T. Hsieh C. Y. (2021). Associations between stroke type, stroke severity, and pre-stroke osteoporosis with the risk of post-stroke fracture: a nationwide population-based study. J. Neurol. Sci. 427, 117512. 10.1016/j.jns.2021.117512 34082148

[B75] Wang D. Wang J. Zhao H. Liang Y. Zhang W. Li M. (2023). The relationship between the prefrontal cortex and limb motor function in stroke: a study based on resting-state functional near-infrared spectroscopy. Brain Res. 1805, 148269. 10.1016/j.brainres.2023.148269 36736871

[B76] Weinstein E. R. Boyer R. B. White R. S. Weinberg R. Y. Lurie J. M. Salvatierra N. (2024). Improved outcomes for spinal versus general anesthesia for hip fracture surgery: a retrospective cohort study of the national surgical quality improvement program. Reg. Anesth. Pain Med. 49 (1), 4–9. 10.1136/rapm-2022-104217 37130697

[B77] Werner N. L. Moore E. E. Hoehn M. Lawless R. Coleman J. R. Freedberg M. (2022). Inflate and pack! pelvic packing combined with REBOA deployment prevents hemorrhage related deaths in unstable pelvic fractures. Injury 53 (10), 3365–3370. 10.1016/j.injury.2022.07.025 36038388

[B78] Xie Z. L. Wang C. C. Huang X. Wang Z. Shangguan H. Y. Wang S. H. (2024). Prevalence and risk factors of stroke in inpatients with type 2 diabetes mellitus in China. Curr. Med. Sci. 44 (4), 698–706. 10.1007/s11596-024-2911-1 39039375

[B79] Yamguchi T. Mori K. Kojima Y. Hasegawa T. Hirota J. Akashi M. (2024). Efficacy of perioperative oral care management in the prevention of surgical complications in 503 patients after pancreaticoduodenectomy for resectable malignant tumor: a multicenter retrospective analysis using propensity score matching. Surgery 175 (4), 1128–1133. 10.1016/j.surg.2023.11.008 38061914

[B80] Yang W. Zhang X. Li Z. Zhang Q. Xue C. Huai Y. (2021). The effect of brain-computer interface training on rehabilitation of upper limb dysfunction after stroke: a meta-analysis of randomized controlled trials. Front. Neurosci. 15, 766879. 10.3389/fnins.2021.766879 35197817 PMC8859107

[B81] Yang H. Li Y. Yang H. Shi Z. Yao Q. Jia C. (2025). A novel CT-Based fracture risk prediction model for COPD patients. Acad. Radiol. 32 (2), 1043–1053. 10.1016/j.acra.2024.08.039 39393992

[B82] Yao H. Junna L. Hu Y. Sha X. Martikainen P. (2023). The relationship of income on stroke incidence in Finland and China. Eur. J. Public Health 33 (3), 360–365. 10.1093/eurpub/ckad035 37087112 PMC10234641

[B83] Yilmaz Eker P. Yilmaz M. (2023). The effect of using a normothermia checklist on awakening time from anesthesia and coagulation disorder: a randomized controlled trial. J. Nurs. Res. 31 (6), e302. 10.1097/jnr.0000000000000583 38015120 PMC11812665

[B84] Youens D. Katzenellenbogen J. Srinivasa Ragavan R. Sodhi-Berry N. Carson J. Zemedikun D. (2023). Differing definitions of first-ever stroke influence incidence estimates more than trends: a study using linked administrative data. Neuroepidemiology 57 (6), 423–432. 10.1159/000534242 37751719

[B85] Zhang N. Guo L. Yu Y. Chen S. Gao L. Hou X. (2023). New-onset stroke on the risk of hip fracture: the kailuan cohort study in China. BMC Public Health 23 (1), 925. 10.1186/s12889-023-15787-5 37217860 PMC10204337

[B86] Zhang B. Zhou H. Wang X. Zheng Y. Hu L. (2024). Advances in the multimodal management of perioperative hypothermia: approaches from traditional Chinese and Western medicine. Perioper. Med. (Lond). 13 (1), 107. 10.1186/s13741-024-00465-w 39472974 PMC11520774

[B87] Zhang G. Zhu G. Hu F. Jiang Y. Li W. Xu D. (2025). Dynamic neural mechanisms underlying sustained motor training post-stroke: an fNIRS investigation employing time-varying ALFF and functional connectivity analysis. J. Biophot. 18 (3), e202400491. 10.1002/jbio.202400491 39832523

[B88] Zhu T. Chen J. Du Y. Li T. Jia X. Lv Y. (2025). Disruptions of resting-state functional connectivity in post-stroke motor dysfunctions: a meta-analysis. Brain Imaging Behav. 19 (3), 771–784. 10.1007/s11682-025-00977-z 40148720

[B89] Zou L. Chen G. Rong Y. Tang C. Lv X. Fan Y. (2025). Three signalling pathways for iron overload in osteoporosis: a narrative review. J. Orthop. Surg. Res. 20 (1), 186. 10.1186/s13018-025-05588-4 39979989 PMC11844007

